# Aspects of functioning and environmental factors in medical work capacity evaluations of persons with chronic widespread pain and low back pain can be represented by a combination of applicable ICF Core Sets

**DOI:** 10.1186/1471-2458-12-1088

**Published:** 2012-12-18

**Authors:** Urban Schwegler, Jessica Anner, Christine Boldt, Andrea Glässel, Veronika Lay, Wout Ernst Lodewijk De Boer, Gerold Stucki, Bruno Trezzini

**Affiliations:** 1 Swiss Paraplegic Research (SPF), Nottwil, Switzerland; 2 asim, Academy of Swiss Insurance Medicine, University Hospital Basel, Basel, Switzerland; 3 Department of Applied Social Sciences, University of Applied Sciences Munich, München, Germany; 4 Department of Health Sciences and Health Policy, University of Lucerne and SPF, Nottwil, Switzerland

**Keywords:** International Classification of Functioning, Disability and Health (ICF), Work capacity evaluation, Chronic widespread pain, Low back pain, Standardization

## Abstract

**Background:**

Medical work capacity evaluations play a key role in social security schemes because they usually form the basis for eligibility decisions regarding disability benefits. However, the evaluations are often poorly standardized and lack transparency as decisions on work capacity are based on a claimant’s disease rather than on his or her functional capacity. A comprehensive and consistent illustration of a claimant’s lived experience in relation to functioning, applying the International Classification of Functioning, Disability and Health (ICF) and the ICF Core Sets (ICF-CS), potentially enhances transparency and standardization of work capacity evaluations. In our study we wanted to establish whether and how the relevant content of work capacity evaluations can be captured by ICF-CS, using disability claimants with chronic widespread pain (CWP) and low back pain (LBP) as examples.

**Methods:**

Mixed methods study, involving a qualitative and quantitative content analysis of medical reports. The ICF was used for data coding. The coded categories were ranked according to the percentage of reports in which they were addressed. Relevance thresholds at 25% and 50% were applied. To determine the extent to which the categories above the thresholds are represented by applicable ICF-CS or combinations thereof, measures of the ICF-CS’ degree of coverage (i.e. content validity) and efficiency (i.e. practicability) were defined.

**Results:**

Focusing on the 25% threshold and combining the Brief ICF-CS for CWP, LBP and depression for CWP reports, the coverage ratio reached 49% and the efficiency ratio 70%. Combining the Brief ICF-CS for LBP, CWP and obesity for LBP reports led to a coverage of 47% and an efficiency of 78%.

**Conclusions:**

The relevant content of work capacity evaluations involving CWP and LBP can be represented by a combination of applicable ICF-CS. A suitable standard for documenting such evaluations could consist of the Brief ICF-CS for CWP, LBP, and depression or obesity, augmented by additional ICF categories relevant for this particular context. In addition, the unique individual experiences of claimants have to be considered in order to assess work capacity comprehensively.

## Background

Even though the process of disability evaluation varies between countries, medical work capacity evaluations usually play a crucial role in deciding on a claimant’s eligibility for benefits provided by national disability insurance schemes. Because of the key role they play, such evaluations ought to be transparent and comprehensible for all persons involved [1-4]. To enhance transparency and comprehensibility, the claimant’s lived experience in relation to his or her functioning as well as with regard to influencing contextual factors should be assessed comprehensively [2,5]. Moreover, the evaluations’ comparability in terms of both interrater reliability between medical experts and content validity is considered as an important quality criterion [6-8]. Finally, standardization is seen as one means to ensure comparability in disability assessments [9,10].

Medical standards usually refer to features which are considered as relevant to a target group in general and less so to individuals’ unique experiences [11,12]. As a basis for comprehensive disability evaluations, however, a suitable standard should also allow the description of relevant experiences unique to the individual, thus complementing the whole process of evaluation [12].

In reality, decisions on work capacity often lack transparency and comprehensibility [10,13-15]. Also, disability assessments are often insufficiently standardized [5,16,17], which affects their content validity and interrater reliability negatively [8,9,17]. In the Swiss national disability insurance scheme, for example, there is no generally accepted tool to guide the structure and content of disability evaluations [3]. Furthermore, decisions on work capacity for certain disorders are partly based on blanket rulings by the Swiss Federal Court [3]. Somatoform pain disorders, for instance, do generally not lead to incapacity for work. Because they are considered to be caused by psychosocial factors, the Swiss Social Security law does not recognize them as a sufficient reason for a disability pension, except if they are accompanied by a psychiatric co-morbidity like, for example, a depressive disorder [18]. By contrast, pain disorders caused by structural impairments (e.g. by a severe intervertebral disc disorder) normally entitle a person to receive disability benefits. However, diagnoses or impairments, are only loosely connected with functional limitations at work [19-21]. Moreover, the World Health Organization defines *impairment* as a loss or abnormality of a psychological, physiological, or anatomical structure or function and *disability* as a restriction or lack of ability to perform an activity in a manner considered to be normal for a human being [22]. Based on these definitions, focusing only on impairments is not sufficient to give a proper statement about a claimant’s functional capacity at work.

Because pain is a subjective sensation, its impact on a claimant’s functional capacity is difficult to objectify. Claimants with somatoform pain disorders could have the same or even a lower functional capacity than persons with a disorder related to a structural impairment. Nevertheless, according to Swiss jurisprudence their work capacity is usually rated higher. With respect to this controversy between the medical and the legal view, it seems crucial to apply a disability-oriented approach and to comprehensively assess the aspects which might influence a claimant’s functioning and health in order to ensure transparent disability evaluations for persons with chronic pain.

Several attempts have been undertaken to enhance transparency and standardization in disability evaluations [23]. The *Guides to the Evaluation of Permanent Impairment* of the *American Medical Association* (AMA) are used for disability and impairment assessment and as a standard for workers’ compensation evaluations in the United States and many English-speaking countries [24]. Furthermore, a number of standardized procedures for work capacity assessments have been developed like, for example, the *Functional Capacity Evaluation* (FCE) [25-27].

FCE, however, is not appropriate for multidisciplinary assessments as it is not geared towards a comprehensive evaluation of the claimant’s functioning. It focuses on physical functional limitations and not on mental functioning [25], and it does not address environmental factors, an important component to ensure transparency in disability evaluations [5,28]. The AMA Guides have been questioned regarding their applicability in disability assessments of claimants with chronic pain [1], because they follow a diagnosis-based and impairment-oriented rather than a disability-oriented approach [29].

As part of the shift in recent years from impairment-oriented to disability-oriented assessments in European social security institutions, it has been suggested that the comprehensive conceptual framework and standardized taxonomy of the *International Classification of Functioning, Disability and Health* (ICF) [30] could improve the disability determination process [16,31-33]. Since the ICF offers a scientific basis for describing results and determinants of functioning, disability and health which also considers contextual factors [30], standardization and transparency in disability evaluations might be enhanced if the taxonomy would be used as a blueprint.

While the ICF framework was generally well-received, the actual application of the taxonomy has been hampered by the sheer number of categories to be assessed, i.e. 362 on the second level and up to 1,424 when applying the more detailed third and fourth levels. Consequently, *ICF Core Sets* (henceforth ICF-CS) have been developed in order to simplify the use of the taxonomy in clinical settings.

ICF-CS preserve the model of the ICF in a useable mode, and they come in two flavors: (1) *brief* ICF-CS include a minimum number of categories describing the most relevant aspects related to functioning in persons with a specific health condition or in a specific setting [34]; (2) *comprehensive* ICF-CS include all categories of the respective brief ICF-CS but also additional ones so as to facilitate multidisciplinary assessments in the clinical context [35].

Because they involve high costs and time resources of medical experts are limited, medical work capacity evaluations should not only be transparent but also efficient and practical [36]. ICF-CS allow to describe a person’s lived experience in a comprehensive and systematic way [35], and might be applied as practical standards regarding *what* should be documented in disability assessments. So far there have been only few attempts to examine the applicability of ICF-CS in disability evaluations [16,37]. To ascertain their potential it is, therefore, vital to provide further empirical evidence.

Currently ICF-CS exist for about 30 health conditions [38]. The ICF-CS for chronic widespread pain (CWP) [39] and low back pain (LBP) [40] were published in 2004 and subsequently validated in the clinical context [41-43]. Due to the high prevalence of disability claims and large social costs based on CWP and LBP [44-47], we chose them as our index conditions. Both conditions are also often diagnosed concurrently [48].

Moreover, CWP has been found to be related to depression [49] and chronic LBP to obesity [50]. Such co-morbidities are routinely addressed in disability assessments of claimants with chronic pain. We, therefore, also included in our analysis the ICF-CS for depression [51] and obesity [52].

### Objective

The objective of the study was to establish whether or not and how the relevant content of medical work capacity evaluations can be captured by ICF-CS, using medical reports from disability claimants with the index conditions CWP and LBP as examples.

### Specific aims

(1) We wanted to examine to what extent the relevant aspects of functioning and environmental factors in medical reports of claimants with CWP and LBP are represented by applicable ICF-CS. (2) We wanted to determine by which ICF-CS, or combinations thereof, these aspects are best represented.

## Methods

### Study design

A mixed methods study [53] was conducted, involving a qualitative and quantitative content analysis [54,55] of medical reports. The ICF was used for data coding.

### Ethics

The study was approved by the Ethics Commission of Basel, Switzerland, project number 134/08, and was performed in accordance with the Declaration of Helsinki.

### Sample

The reports analyzed were derived from an elicitation of all medical reports received by the major Swiss health and accident insurers between February 1 and April 31, 2008, as part of a study on the quality of medical work capacity evaluations in Switzerland [3]. Insurance employees selected and anonymized all reports containing a diagnosis of CWP and/or LBP based on the *International Classification of Diseases* (ICD-10) (see Table 1). The diagnoses were checked by two health professionals. To ensure comparability, only reports in German submitted to the Swiss national disability insurance scheme were selected. Reports in French and Italian as well as from accident, health and liability insurances were excluded.

**Table 1:** ICD-10 diagnoses included in the sample

**ICD-10 diagnoses for CWP**		**ICD-10 diagnoses for LBP**	
F45.0	Somatization disorder	M42	Spinal osteochondrosis (.15-.17, .95-.97)
F45.1	Undifferentiated somatoform disorder	M45	Ankylosing spondylitis
F45.4	Persistent somatoform disorder	M46	Other inflammatory spondylopathies (.0, .1, .2, .3)
F54	Psychological and behavioral factors associated with disorders or diseases classified elsewhere	M47	Spondylosis and (osteo-)arthrosis of spine (.05-.07, .15-.17, .25-.27)
F62.8	Chronic pain personality syndrome	M48	Other spondylopathies (.05-.07, .15-.17, .25-.27)
F32	Mild, moderate and severe depressive episode, with somatic symptoms	M51	Other intervertebral disc disorders (.0, .1)
F33	Recurrent depressive disorder, with somatic symptoms	M53	Other dorsopathies, not elsewhere classified (.25-.27, .3, .86-.87, .96-.97)
F34.1	Dysthymia (in relation with pain)	M54	Dorsalgias (.05-.07, .15-.17, .3, .4, .5, .85-.87)
F43.2	Adjustment disorders	M99	Biomechanical lesions, not elsewhere classified (.03, .13, .23, .33, .43, .53, .63, .73, .83, .93)
M79.7	Fibromyalgia		
R52.2	Other chronic pain		
R52.9	Pain, unspecified		

From this basic sample a subsample was randomly drawn. The determination of the final sample size was based on two criteria: (1) *heterogeneity*, i.e. the relevant medical disciplines of pain-assessment and the index conditions (CWP, LBP) were to be included proportionally; and (2) *saturation*, i.e. the collected information was considered to be sufficient when no new second-level ICF category emerged in five successive reports analyzed [56-58]. In order to satisfy the heterogeneity requirement, i.e. a proportional inclusion of the medical disciplines and the index conditions, a minimum size of the subsample was determined.

### Analysis plan

For the data analysis the sample was divided into two sub-groups: (1) reports with CWP diagnoses, and (2) reports with LBP diagnoses. Reports including both diagnoses entered the data analysis twice, once with the pure CWP and once with the pure LBP reports.

To examine the extent to which the relevant aspects of functioning and environmental factors in medical reports of claimants with CWP and LBP are represented by applicable ICF-CS, we first did a content analysis of the reports, using the ICF for data coding. We then ranked the coded categories for both sub-groups according to their relevance, i.e. their relative frequency across reports, setting thresholds at 25% and 50%. Next, we examined whether the relevant ICF categories in CWP reports, i.e. the ones above the thresholds, are represented by the ICF-CS for the index condition (CWP) and major co-morbidities (LBP, depression). For LBP reports, we did the same analysis with the ICF-CS for the index condition (LBP) and major co-morbidities (CWP, obesity). By calculating and comparing values for their coverage (i.e. their content validity) and efficiency (i.e. their potential practicability) we determined to what extent the relevant aspects in the reports are represented by the ICF-CS for the index-condition, the co-morbidities, and a combination thereof and which ICF-CS or combination thereof is best representing these aspects.

### Analysis

#### Content analysis

Our raw data consisted of reports on disability claimants. They comprised one or more medical disciplines and included information on: (a) socio-medical history, (b) medical examination, and (c) work capacity evaluation. This content was coded to the ICF by applying established linking rules [59,60].

The reports were dissected into text passages, each representing a self-contained *unit of meaning* (e.g. “the claimant suffers from pain while walking”). The various *concepts* underlying a unit of meaning were determined (e.g. pain, walking) and coded to the most precise ICF *category* (e.g. b280 Sensation of pain, d450 Walking) by two health professionals trained in the ICF. A concept could be linked to more than one ICF category. Each instance of a category code being assigned to a concept was referred to as a *coding*. Concepts not appropriately codeable to ICF categories were flagged as either *personal factors* (e.g. individual attitudes and beliefs), *not covered* (e.g. degree of disability), *not definable* (e.g. demanding activities), or *health condition* (e.g. diabetes). The two coders assessed whether the categories represented *limitations* (e.g. “the claimant suffers from back pain”) or, in case that they were environmental factors, whether they were *barriers* (e.g. “the surgery made the pain worse”) or *facilitators* (e.g. “the surgery was helpful”) for the claimant, were *no problem* (e.g. “the surgery had no effect”), or *facts* (e.g. “the surgery was performed recently”). Finally, the coders had to agree on the chosen codes. Any disagreement was solved in consultation with a third person experienced in the linking method.

#### Reliability and saturation

The interrater agreement was calculated using Cohen’s kappa coefficient [61]. The saturation level was checked after each additional report analyzed.

#### Relevance ranking

Referring to the *absolute frequency* for determining relevance was deemed potentially misleading because different writing styles of medical experts could have led to varying degrees of content repetitions. Therefore, we operationalized the *relevance* of a coded category as its *relative frequency* across reports, i.e. the percentage of reports in which it appeared as a *limitation*, *barrier* or *facilitator* for the claimant. In order to ensure comparability with the ICF-CS, which refer to aspects that are problematic or supportive for the patients, we did not include the ICF categories assessed as *no problem* or *facts* in the ranking. Moreover, since the concepts not appropriately codeable with the ICF were not further specified in this study, they were not included in the ranking. Thus, the final ranking involved only second-level ICF categories coded either as a limitation, barrier or facilitator. For the ensuing data analysis we defined two *thresholds of minimum relevance*, the more lenient one at 25% or more of the reports, the more stringent one at 50% or more.

#### Coverage and efficiency ratios

We used two criteria to examine the extent to which the relevant content of medical reports involving CWP and LBP is represented by ICF-CS. (1) The *coverage ratio*, i.e. the ability of ICF-CS to capture the relevant aspects of the context in which they are applied (namely the index conditions CWP and LBP and the assessment of work capacity as part of disability evaluations). It was calculated as the number of ICF-CS categories above the threshold of 25% (or 50%) divided by the total number of ICF categories above the threshold. (2) The *efficiency ratio*, i.e. the ability of ICF-CS to be manageable and to contain only as many categories as necessary. It was calculated as the number of ICF-CS categories above the threshold divided by the total number of categories in the ICF-CS. A definition of efficiency which is similar to ours was applied in a recent study where it was defined as the ability of a measurement instrument to be manageable and to contain as few items as possible that measure variables outside a domain set of ICF categories used in that study [62].

ICF-CS should ideally show a high coverage ratio and be efficient at the same time.

**Figure 1:**
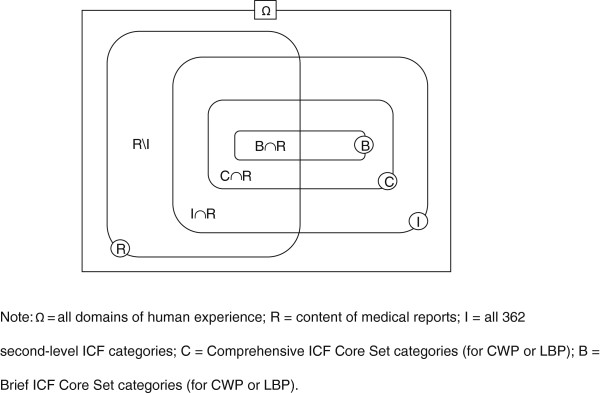
Operationalization of an ICF Core Set’s coverage and efficiency ratios.

Referring to Figure 1, the operationalization of the coverage and efficiency ratios can be further illustrated as follows:

$$\text{Coverage}\;\text{ratio}\;\left(\text{Brief}\;\text{ICF}–\text{CS}\right)=\left(B\cap R\right)/\left(I\cap R\right)$$

$$\begin{array}{l} \text{Coverage}\;\text{ratio}\;\left(\text{Comprehensive}\;\text{ICF}–\text{CS}\right)\\ \;=\left(C\cap R\right)/\left(I\cap R\right) \end{array}$$

$$\text{Efficiency}\;\text{ratio}\;\left(\text{Brief}\;\text{ICF}–\text{CS}\right)=B\cap R/B$$

$$\begin{array}{l} \text{Efficiency}\;\text{ratio}\;\left(\text{Comprehensive}\;\text{ICF}–\text{CS}\right)\\ \;=\left(C\cap R\right)/C \end{array}$$

## Results

### Sample characteristics

In order to satisfy the heterogeneity requirement, the required minimum sample size had been determined to be 72 medical reports, representing about one third of the basic sample of 209 reports. The saturation criterion was already reached after coding 30 reports. The number and type of disciplines in the reports are displayed in Table 2.

**Table 2:** Medical disciplines represented in the reports

	**CWP**	**LBP**
**Number of medical disciplines in report**		
One	20	14
Two	4	5
More than two	26	26
**Medical discipline**		
Psychiatry	45	31
Rheumatology	21	22
Internal medicine	16	16
Neurology	10	11
Orthopedics	9	12
General medicine	11	9
Neurosurgery	1	5
Orthopedic surgery	1	3
Neuropsychology	1	3
Pneumology	1	-
Hand surgery	1	1
Functional capacity evaluation	-	1

27 reports contained only a CWP diagnosis, 22 only a LBP diagnosis, and 23 both a CWP and LBP diagnosis. Of the 50 reports with CWP diagnoses, 24 (48%) also included a diagnosis of the ICD-10-four-character subcategory “Mood [affective] disorders”. Of the 45 reports with LBP diagnoses, 13 (29%) additionally involved a diagnosis related to “Obesity and other hyperalimentation”.

The overall interrater agreement (Cohen’s kappa) at the second ICF-level was 0.80 (0.79 - 0.81; 95% bootstrap confidence interval [63]).

### Reports with CWP diagnoses

#### Content analysis

21,562 units of meaning gave rise to 45,365 (100%) codings. 30,042 (66.2%) represented links to ICF categories. The remainder (15,323 or 33.8%), i.e. R/I in Figure 1, were not classifiable appropriately with the ICF. Of these, 4,276 (9.4%) codings represented *personal factors*, the as yet unspecified fifth component of the ICF. 4,094 (9%) codings were labeled as *not covered*, 4,710 (10.4%) as *not definable*, and 2,243 (4.9%) as *health condition*.

#### Relevance ranking

76 ICF categories passed the 25% and 37 the 50% threshold and were identified as relevant for CWP reports. Table 3 shows if the categories are included in the ICF-CS for CWP, LBP and depression.

**Table 3:** Relative frequency ranking of the ICF categories found in the CWP reports (n = 50)

**Rank**	**ICF code**	**ICF category**	**CWP**		**LBP**		**Depression**		**Relative frequency (%)**	**Absolute frequency**
			**Brief (k=24)**	**Compr. (k=67)**	**Brief (k=35)**	**Compr. (k=78)**	**Brief (k=31)**	**Compr. (k=121)**		
1	b280	Sensation of pain	X	X	X	X	.	X	100	2531
2	b152	Emotional functions	X	X	X	X	X*	X	98	640
3	b130	Energy and drive functions	X	X	X	X	X*	X	98	393
4	d850	Remunerative employment	X	X	X	X	.	X	96	344
5	b126	Temperament and personality functions	.	X	.	X	X*	X	94	445
6	b134	Sleep functions	X	X	X	X	.	X	92	222
7	e310	Immediate family	X	X	X	X	X	X	90	332
	e110†	Products or substances for personal consumption	X*	X*	X	X	X*	X*	90	184
8	e580	Health services, systems and policies	.	X	X	X	X	X	88	106
9	d240	Handling stress and other psychological demands	X	X	X	X	X	X	86	177
10	d570	Looking after one’s health	.	X	.	X	X	X	86	154
11	b270	Sensory functions related to temperature and other stimuli	.	X	.	.	.	.	82	225
12	e1101	Drugs	X	X	X*	X*	X	X	82	140
	b160†	Thought functions	X*	X*	.	.	.	X	80	337
13	b730	Muscle power functions	X	X	X	X	.	.	78	180
14	b710	Mobility of joint functions	.	X	X	X	.	.	74	365
15	b1602	Content of thought	X	X	.	.	.	X	74	145
16	e570	Social security services, systems and policies	X	X	X	X	.	X	74	130
17	s760	Structure of trunk	.	.	X	X	.	.	70	571
18	d415	Maintaining a body position	.	X	X	X	.	.	70	201
19	e165	Assets	.	.	.	.	.	X	70	89
20	d450	Walking	X	X	X	X	.	.	68	141
21	d760	Family relationships	X	X	X	X	X	X	68	103
22	d230	Carrying out daily routine	X	X	.	.	X*	X	68	98
23	b435	Immunological system functions	.	.	.	.	.	.	64	207
24	b735	Muscle tone functions	.	X	X	X	.	.	64	122
25	d430	Lifting and carrying objects	X	X	X	X	.	.	64	104
26	b455	Exercise tolerance functions	X	X	X	X	.	.	64	102
27	d870	Economic self-sufficiency	.	.	.	.	.	X	64	73
28	d920	Recreation and leisure	X	X	.	X	.	X	64	66
29	d770	Intimate relationships	X	X	.	X	X	X	62	74
30	d410	Changing a basic body position	.	X	X	X	.	.	58	84
31	d750	Informal social relationships	.	.	.	.	.	X	58	53
32	s750	Structure of lower extremity	.	.	.	X	.	.	56	179
33	d845	Acquiring, keeping and terminating a job	.	X	X	X	X	X	56	68
34	b140	Attention functions	.	X	.	.	X	X	56	60
35	b147	Psychomotor functions	X	X	.	.	X	X	54	80
36	b144	Memory functions	.	.	.	.	.	X	52	65
37	b530	Weight maintenance functions	.	.	.	.	.	X	50	86
38	e565	Economic services, systems and policies	.	.	.	.	.	.	48	50
39	e410	Individual attitudes of immediate family members	X	X	X	X	X	X	46	72
40	e225	Climate	.	.	.	X	.	X	44	53
41	d720	Complex interpersonal interactions	.	X	.	.	.	X	44	45
42	d160	Focusing attention	.	X	.	.	.	.	44	44
43	d475	Driving	.	X	.	X	.	X	44	38
44	b240	Sensations associated with hearing and vestibular function	.	.	.	.	.	.	42	47
45	b810	Protective functions of skin	.	.	.	.	.	.	42	39
46	d445	Hand and arm use	.	.	.	X	.	.	40	56
47	b420	Blood pressure functions	.	.	.	.	.	.	40	44
48	d350	Conversation	.	.	.	.	X	X	40	32
49	b460	Sensations associated with cardiovascular and respiratory functions	.	.	.	.	.	X	38	44
50	s720	Structure of shoulder region	.	.	.	.	.	.	38	43
51	b110	Consciousness functions	.	.	.	.	.	.	38	40
52	e325	Acquaintances, peers, colleagues, neighbours and community members	.	X	.	X	X	X	38	28
53	e315	Extended family	.	.	.	.	.	.	36	31
54	d440	Fine hand use	.	.	.	.	.	.	34	52
55	b620	Urination functions	.	.	.	X	.	.	34	42
56	b535	Sensations associated with the digestive system	.	.	.	.	.	X	34	34
57	e120	Products and technology for personal indoor and outdoor mobility and transportation	.	.	.	X	.	.	32	67
58	d640	Doing housework	X	X	X	X	.	X	32	35
59	e245	Time-related changes	.	.	.	.	.	X	32	35
60	b780	Sensations related to muscles and movement functions	.	X	.	X	.	X	32	33
61	b415	Blood vessel functions	.	.	.	.	.	.	32	31
62	b510	Ingestion functions	.	.	.	.	.	.	32	24
63	d166	Reading	.	.	.	.	.	X	32	16
64	b525	Defecation functions	.	.	.	.	.	.	30	33
65	b770	Gait pattern functions	.	.	.	X	.	.	30	31
66	s740	Structure of pelvic region	.	.	.	X	.	.	30	30
67	d660	Assisting others	.	X	.	X	.	X	28	27
68	s120	Spinal cord and related structures	.	.	X	X	.	.	28	27
69	b750	Motor reflex functions	.	.	.	X	.	.	28	26
70	d540	Dressing	.	X	X	X	.	X	28	25
71	e355	Health professionals	X	X	X	X	X	X	28	23
72	d455	Moving around	.	X	.	X	.	.	28	20
73	e320	Friends	.	.	.	.	X	X	28	18
74	d740	Formal relationships	.	.	.	.	.	.	26	33
75	b164	Higher-level cognitive functions	.	X	.	.	.	X	26	25
76	b830	Other functions of the skin	.	.	.	.	.	.	26	20
77	s730	Structure of upper extremity	.	.	.	.	.	.	24	45
78	e430	Individual attitudes of people in positions of authority	.	X	.	.	.	X	24	37
79	d460	Moving around in different locations	.	.	.	X	.	.	24	27
80	e510	Services, systems and policies for the production of consumer goods	.	.	.	.	.	.	24	24
81	d710	Basic interpersonal interactions	.	.	.	X	.	X	24	23
82	d950	Political life and citizenship	.	.	.	.	.	X	24	21
83	b640	Sexual functions	.	X	.	X	.	X	24	20
84	e115	Products and technology for personal use in daily living	.	.	.	.	.	.	24	19
85	d330	Speaking	.	.	.	.	.	X	24	19
86	s320	Structure of mouth	.	.	.	.	.	.	24	16
87	d620	Acquisition of goods and services	.	X	.	X	.	X	24	14
**Ranking of the remaining categories of the Brief ICF Core Sets for CWP, LBP and depression:**										
92	b740	Muscle endurance functions	.	X	X	X	.	.	22	17
95	e450	Individual attitudes of health professionals	.	X	X	X	X	X	20	14
98	e135	Products and technology for employment	.	.	X	X	.	.	18	18
99	s770	Additional musculoskeletal structures related to movement	.	X	X	X	.	.	18	13
100	e550	Legal services, systems and policies	.	.	X	X	.	.	18	12
103	d859	Work and employment, other specified and unspecified	.	.	X	X	.	.	16	20
116	d163	Thinking	.	.	.	.	X	X	14	9
117	b760	Control of voluntary movement functions	X	X	.	.	.	.	14	8
121	b715	Stability of joint functions	.	.	X	X	.	.	14	7
139	e415	Individual attitudes of extended family members	.	.	.	.	X	X	8	11
161	d530	Toileting	.	.	X	X	.	.	6	4
185	d510	Washing oneself	.	X	.	X	X	X	4	2
210	d175	Solving problems	X	X	.	.	X	X	2	1
212	e420	Individual attitudes of friends	.	X	.	.	X	X	2	1
221	d177	Making decisions	.	.	.	.	X	X	2	1

Note: k = total number of categories in the respective ICF Core Set; † = ICF categories that were ignored in the ranking because the Brief and Comprehensive ICF Core Sets for CWP contain them on the more specific third level; X = included in the particular ICF Core Set (CWP, LBP or depression); * = in the particular ICF Core Set the stated category is included at the next lower (third) or next higher (second) level.

#### Coverage and efficiency ratios

Focusing on the more inclusive 25% threshold, the relevant aspects of functioning and environmental factors in CWP reports are represented with a coverage of 29% [54%] and an efficiency of 92% [61%] by the Brief [Comprehensive] ICF-CS for CWP (see Table 4).

**Table 4:** Coverage and efficiency ratios of the different ICF Core Sets for the CWP-reports (n = 50) and the two relevance thresholds

	**Number of overlapping categories**	**Coverage ratio (%)**	**Efficiency ratio (%)**
**Relevance threshold ≥ 25% (m = 76)**			
CWP Brief (k = 24)	22	29	92
CWP Comprehensive (k = 67)	41	54	61
LBP Brief (k = 35)	29	38	83
LBP Comprehensive (k = 78)	43	57	55
Depression Brief (k = 26†)	19	25	73
Depression Comprehensive (k = 90†)	43	57	48
CWP + LBP + Depression Brief (k = 53*)	37	49	70
CWP + LBP + Depression Comprehensive (k = 131*)	62	82	47
**Relevance threshold ≥ 50% (m = 37)**			
CWP Brief (k = 24)	19	51	79
CWP Comprehensive (k = 67)	29	78	43
LBP Brief (k = 35)	21	57	60
LBP Comprehensive (k = 78)	26	70	33
Depression Brief (k = 26†)	14	38	54
Depression Comprehensive (k = 90†)	26	70	29
CWP + LBP + Depression Brief (k = 53†*)	29	78	55
CWP + LBP + Depression Comprehensive (k = 131†*)	36	97	27

Note: m = total number of ranked categories above the respective threshold; k = total number of categories in the respective ICF Core Set; † = categories aggregated on the second level (expect categories only available on the third level in the Comprehensive ICF Core Set); * = adjusted for overlap between the categories of the three ICF Core Sets.

When combining the ICF-CS for CWP, LBP and depression, the coverage ratio of the Brief [Comprehensive] ICF-CS was with 49% [82%] substantially higher and the efficiency ratio with 70% [47%] lower compared to the ICF-CS for CWP.

### Reports with LBP diagnoses

#### Content analysis

21,707 units of meaning led to 42,116 (100%) codings. 28,876 (68.6%) represented ICF categories. Of the 13,240 (31.4%) codings not classifiable appropriately with the ICF, 3,111 (7.4%) were labeled as *personal factors*, 3,322 (7.9%) as *not covered*, 4,236 (10.1%) as *not definable*, and 2,571 (6.1%) as *health condition*.

#### Relevance ranking

74 ICF categories passed the 25% and 33 the 50% threshold and were identified as relevant for LBP reports. Table 5 shows if the categories are included in the ICF-CS for LBP, CWP and obesity.

**Table 5:** Relative frequency ranking of the ICF categories found in the LBP reports (n = 45)

**Rank**	**ICF Code**	**ICF Category**	**LBP**		**CWP**		**Obesity**		**Relative Frequency (%)**	**Absolute Frequency**
			**Brief (k=35)**	**Compr. (k=78)**	**Brief (k=24)**	**Compr. (k=67)**	**Brief (k=8)**	**Compr. (k=109)**		
1	b280	Sensation of pain	X	X	X	X	.	X	100	2462
2	d415	Maintaining a body position	X	X	.	X	.	X	100	289
3	s760	Structure of trunk	X	X	.	.	.	X	98	958
4	b710	Mobility of joint functions	X	X	.	X	.	X	98	490
5	d850	Remunerative employment	X	X	X	X	.	X	91	325
6	b730	Muscle power functions	X	X	X	X	.	.	91	192
7	b270	Sensory functions related to temperature and other stimuli	.	.	.	X	.	.	87	260
8	d450	Walking	X	X	X	X	X	X	87	158
9	b735	Muscle tone functions	X	X	.	X	.	.	87	119
10	b134	Sleep functions	X	X	X	X	.	X	84	157
11	d430	Lifting and carrying objects	X	X	X	X	.	X	84	151
12	d570	Looking after one’s health	.	X	.	X	X	X	82	122
13	b152	Emotional functions	X	X	X	X	.	X	80	446
14	b126	Temperament and personality functions	.	X	.	X	.	X	80	335
15	b130	Energy and drive functions	X	X	X	X	X	X	80	277
16	d410	Changing basic body position	X	X	.	X	.	X	80	111
17	e110	Products or substances for personal consumption	X	X	X*	X*	X	X	78	188
18	e580	Health services, systems and policies	X	X	.	X	.	X	76	101
19	e310	Immediate family	X	X	X	X	X	X	73	171
20	b435	Immunological system functions	.	.	.	.	.	X	71	171
21	e570	Social security services, systems and policies	X	X	X	X	.	X	69	97
22	s750	Structure of lower extremity	.	X	.	.	.	X	64	275
23	b530	Weight maintenance functions	.	.	.	.	X	X	64	81
24	e165	Assets	.	.	.	.	.	.	64	57
25	b160	Thought functions	.	.	X*	X*	.	.	62	202
26	d240	Handling stress and other psychological demands	X	X	X	X	X	X	62	137
27	d920	Recreation and leisure	.	X	X	X	.	X	62	73
28	d230	Carrying out daily routine	.	.	X	X	.	.	60	90
29	b420	Blood pressure functions	.	.	.	.	.	X	60	40
30	d870	Economic self-sufficiency	.	.	.	.	.	X	58	55
31	d760	Family relationships	X	X	X	X	.	X	56	64
32	d845	Acquiring, keeping and terminating a job	X	X	.	X	.	X	53	40
33	b455	Exercise tolerance functions	X	X	X	X	.	X	51	57
34	s720	Structure of shoulder region	.	.	.	.	.	.	49	48
35	e225	Climate	.	X	.	.	.	X	47	52
36	d445	Hand and arm use	.	X	.	.	.	.	44	49
37	b750	Motor reflex functions	.	X	.	.	.	.	44	43
38	d750	Informal social relationships	.	.	.	.	.	X	44	38
39	d455	Moving around	.	X	.	X	X	X	42	38
40	d770	Intimate relationships	.	X	X	X	.	X	42	35
41	b147	Psychomotor functions	.	.	X	X	.	.	40	60
42	b770	Gait pattern functions	.	X	.	.	.	.	40	42
43	b144	Memory functions	.	.	.	.	.	.	38	61
44	e565	Economic services, systems and policies	.	.	.	.	.	.	38	44
45	d440	Fine hand use	.	.	.	.	.	.	36	50
46	b140	Attention functions	.	.	.	X	.	.	36	49
47	e245	Time-related changes	.	.	.	.	.	.	36	35
48	s740	Structure of pelvic region	.	X	.	.	.	.	36	34
49	b415	Blood vessel functions	.	.	.	.	.	X	36	28
50	d350	Conversation	.	.	.	.	.	.	36	25
51	b810	Protective functions of the skin	.	.	.	.	.	.	36	22
52	s120	Spinal cord and related structures	X	X	.	.	.	.	33	37
53	b620	Urination functions	.	X	.	.	.	X	33	25
54	s730	Structure of upper extremity	.	.	.	.	.	.	31	72
55	b240	Sensations associated with hearing and vestibular functions	.	.	.	.	.	.	31	37
56	d160	Focusing attention	.	.	.	X	.	.	31	35
57	d640	Doing housework	X	X	X	X	.	X	31	30
58	d475	Driving	.	X	.	X	.	X	31	29
59	d540	Dressing	X	X	.	X	.	X	31	27
60	b755	Involuntary movement reaction functions	.	.	.	.	.	.	31	27
61	b715	Stability of joint functions	X	X	.	.	.	.	31	26
62	d720	Complex interpersonal interactions	.	.	.	X	.	.	31	24
63	e325	Acquaintances, peers, colleagues, neighbours and community members	.	X	.	X	.	X	31	22
64	b525	Defecation functions	.	.	.	.	.	.	31	20
65	e315	Extended family	.	.	.	.	.	.	29	26
66	e115	Products and technology for personal use in daily living	.	.	.	.	.	X	29	21
67	b535	Sensations associated with the digestive system	.	.	.	.	.	X	27	30
68	e410	Individual attitudes of immediate family members	X	X	X	X	.	X	27	25
69	b460	Sensations associated with cardiovascular and respiratory functions	.	.	.	.	.	.	27	24
70	b780	Sensations related to muscles and movement functions	.	X	.	X	.	.	27	23
71	b740	Muscle endurance functions	X	X	.	X	.	.	27	19
72	e430	Individual attitudes of people in positions of authority	.	.	.	X	.	.	27	18
73	b640	Sexual functions	.	X	.	X	.	X	27	15
74	d166	Reading	.	.	.	.	.	.	27	15
75	e120	Products and technology for personal indoor and outdoor mobility and transportation	.	X	.	.	.	X	24	62
76	b164	Higher-level cognitive functions	.	.	.	X	.	.	24	43
77	e510	Services, systems and policies for the production of consumer goods	.	.	.	.	.	X	24	26
78	b110	Consciousness functions	.	.	.	.	.	.	22	20
79	s320	Structure of mouth	.	.	.	.	.	.	22	19
80	b755	Involuntary movement functions	.	.	.	.	.	.	22	18
81	d620	Acquisition of goods and services	.	X	.	X	.	X	22	13
82	s770	Additional musculoskeletal structures related to movement	X	X	.	X	.	X	22	12
**Ranking of the remaining categories of the Brief ICF Core Sets for LBP, CWP and obesity:**										
89	e355	Health professionals	X	X	X	X	.	X	20	11
92	d859	Work and employment, other specified and unspecified	X	X	.	.	.	.	18	20
94	e135	Products and technology for employment	X	X	.	.	.	.	18	15
103	e450	Individual attitudes of health professionals	X	X	.	X	.	X	16	13
104	b760	Control of voluntary movement functions	.	.	X	X	.	.	16	13
105	e155	Design, construction and building products and technology of buildings for private use	X	X	.	.	.	X	16	11
122	e550	Legal services, systems and policies	X	X	.	.	.	.	11	13
201	d175	Solving problems	.	.	X	X	.	.	2	1
-	d530	Toileting	X	X	.	.	.	X	0	0

Note: k = total number of categories in the respective ICF Core Set; X = included in the particular ICF Core Set (LBP, CWP or obesity); * = in the particular ICF Core Set the stated category is included at the next lower (third) or next higher (second) level.

#### Coverage and efficiency ratios

Focusing on the 25% threshold, the relevant aspects of functioning and environmental factors in LBP reports are represented with a coverage of 36% [58%] and an efficiency of 77% [55%] by the Brief [Comprehensive] ICF-CS for LBP (see Table 6).

**Table 6:** Coverage and efficiency ratios of the different ICF Core Sets for the LBP-reports (n = 45) and the two relevance thresholds

	**Number of overlapping categories**	**Coverage ratio (%)**	**Efficiency ratio (%)**
**Relevance threshold ≥ 25% (m = 74)**			
LBP Brief (k = 35)	27	36	77
LBP Comprehensive (k = 78)	43	58	55
CWP Brief (k = 24)	21	28	88
CWP Comprehensive (k = 67)	41	55	61
Obesity Brief (k = 8)	8	11	100
Obesity Comprehensive (k = 108†)	41	55	38
LBP + CWP + Obesity Brief (k = 45†*)	35	47	78
LBP + CWP + Obesity Comprehensive (k = 143†*)	59	80	41
**Relevance threshold ≥ 50% (m = 33)**			
LBP Brief (k = 35)	21	64	60
LBP Comprehensive (k = 78)	25	76	32
CWP Brief (k = 24)	17	52	71
CWP Comprehensive (k = 67)	26	79	39
Obesity Brief (k = 8)	7	21	88
Obesity Comprehensive (k = 108†)	27	82	25
LBP + CWP + Obesity Brief (k = 45†*)	26	79	58
LBP + CWP + Obesity Comprehensive (k = 143†*)	32	97	22

Note: m = total number of ranked categories above the respective threshold; k = total number of categories in the respective ICF Core Set(s); † = categories aggregated on the second level; * = adjusted for overlap between the categories of the three ICF Core Sets.

When combining the ICF-CS for CWP, LBP and obesity, the coverage ratio of the Brief [Comprehensive] ICF-CS was with 47% [80%] substantially higher and the efficiency ratio with 78% [41%] lower compared to the ICF-CS for LBP.

## Discussion

We found that the relevant content of medical work capacity evaluations involving CWP and LBP can be captured to a considerable, albeit not perfect, extent by a combination of applicable ICF-CS. The relevant aspects of functioning and environmental factors in the reports were either represented by the ICF-CS for the index conditions (CWP, LBP) or for major co-morbidities (depression, obesity). In both groups of reports and for both relevance thresholds, a combination of the ICF-CS analyzed showed substantially higher coverage ratios than the condition-specific ICF-CS, i.e. they represented the relevant aspects of medical work capacity evaluations involving CWP and LBP to a higher extent. There is, however, a trade-off. Due to the increased number of categories when combining the ICF-CS, the efficiency ratios decreased considerably compared to the condition-specific ICF-CS in most cases.

An interesting finding with regard to the medical disciplines involved in the medical reports was that, in fact, psychiatry appeared in both groups of reports as the most frequent discipline. This clearly indicates the relevance of psychiatric assessments for multidisciplinary medical work capacity evaluations of persons with CWP and LBP and is also in line with the finding that a considerable percentage of our medical reports included a co-morbid disorder from the ICD-10 chapter “Mood [affective] disorders”.

Overall, our results are in line with previous research in the field which found that the Comprehensive ICF-CS for CWP and LBP have a potential for structuring work capacity assessments [37].

Our findings are also in agreement with the recently developed *ICF Core Sets for vocational rehabilitation*[64] regarding the importance of highlighting the components activities, participation and environmental factors in the context of work and work capacity.

Finally, with regard to the *generic core set for disability evaluation in social security*[32] we feel that its lack of environmental factors may be a potential limitation if one aims for a comprehensive and transparent documentation of a claimant’s work capacity. While the authors argue that environmental aspects are implicitly covered by the participation items, we found in our analysis of medical reports prepared in the context of disability evaluations that a number of environmental factors (e.g. e310 Immediate family; e165 Assets) are explicitly and frequently reported as barriers or facilitators for the claimants (see Tables 3 and 5).

### Study limitations

Our study has some limitations. Our sample only included medical reports in German of the Swiss national disability insurance scheme with an ICD-10-diagnosis for CWP and/or LBP. The results may therefore not be generalizable to other health conditions, nor to other insurance schemes or other countries with different disability evaluation procedures. Future research should involve validation studies which look into the generalizability of our findings.

Another limitation was the significant amount of content not appropriately addressed in the current ICF taxonomy. This refers mainly to some specific aspects of functioning related to work capacity (e.g. demanding activities) and to personal factors, which may influence work capacity [65] and could, when explicitly addressed, contribute to more transparent disability evaluations [66]. This limitation could have potentially missed factors critical and relevant to the process of work capacity evaluation which should be taken into account in future research.

Finally, one could argue that context-specific ICF-CS relevant to the field of work capacity evaluation, like the ones for vocational rehabilitation or the generic core set for disability evaluation in social security, may have been included in our analysis as well. However, as our sample included medical reports with the index conditions CWP and LBP, we decided to focus rather on condition-specific ICF-CS than on context-specific or generic ones. It might be an issue for further research to determine the extent to which these ICF-CS are representing the content of medical reports of disability claimants.

### Practical implications

Combining ICF-CS (e.g. CWP with LBP and depression, or LBP with CWP and obesity) is a more effective approach for work capacity evaluations involving CWP and LBP than using solely condition-specific ICF-CS. Taken together, the ICF-CS show a potential for guiding comprehensive multidisciplinary assessments. In particular, they could ensure transparency in disability evaluations as well as standardize them in terms of what should be documented. However, efficiency and practicability become problematic when simply combining ICF-CS due to the high number of categories to be assessed. To ensure high coverage *and* efficiency, a suitable standard for medical work capacity evaluations involving CWP and LBP could include:

(1) All categories of the Brief ICF-CS for the index conditions and major co-morbidities because Brief ICF-CS are considered as a minimum standard or data set to be reported in different settings so as to enhance comparability [35];

(2) Those categories of the Comprehensive ICF-CS identified as relevant for the present context;

(3) Those categories not included in the ICF-CS but identified as relevant for the present context (e.g. b435 Immunological system functions for CWP reports; e165 Assets for LBP reports).

Our relevance rankings display the categories which should be included in the standard. To ensure comprehensive evaluations, we recommend to focus on categories above the 25% threshold. Before being applied, however, future research would have to focus on a validation of the categories by experts in the field of work capacity evaluation.

Furthermore, the proposed ICF categories are the basis for a transparent documentation of those aspects of functioning which are relevant for a claimant’s work capacity and should be seen as a complement to the claimant’s diagnosis without necessarily having a direct implication on the work capacity decision itself. Whereas the categories can be used as a guideline for the evaluations in terms of *what* aspects should be documented, they are not addressing the issue of how these aspects should be *assessed*. This latter problem could be approached by assigning existing validated rating instruments to the suggested ICF categories.

Last but not least, it is important to emphasize that aspects of functioning which refer to the unique individual experience of a claimant, but are not necessarily addressed by the abovementioned ICF categories, should be considered in addition as complementary source of information to provide a comprehensive picture of the claimant.

## Conclusions

The relevant content of medical work capacity evaluations involving CWP and LBP can be represented to a considerable extent by a combination of the ICF-CS for the index conditions and major co-morbidities. A suitable approach for a standardized documentation of the evaluations and for enhancing their transparency could consist of the Brief ICF-CS, augmented by additional ICF categories relevant for this particular context. Aspects not appropriately addressed in the current ICF taxonomy, such as personal factors, should be specified and eventually incorporated in such a standard as well. In addition, the unique individual experiences of claimants have to be taken into account in order to assess work capacity comprehensively.

## Competing interests

The authors declare that they have no competing interests.

## Authors’ contributions

Urban Schwegler and Bruno Trezzini prepared the first draft of this paper. All other co-authors made substantial comments on the content of this manuscript. All authors read and approved the final manuscript.

## Pre-publication history

The pre-publication history for this paper can be accessed here:

http://www.biomedcentral.com/1471-2458/12/1088/prepub
